# Case-report: EBV driven lymphoproliferative disorder associated with Ruxolitinib

**DOI:** 10.1186/s12878-015-0029-1

**Published:** 2015-07-11

**Authors:** Róbert Pálmason, Ola Lindén, Johan Richter

**Affiliations:** Department of Hematology and vascular disorders, Skåne University Hospital, Lund, Sweden; Department of Oncology, Skåne University Hospital, Lund, Sweden; Lund University, Lund, Sweden

**Keywords:** Ruxolitinib, Side-effects, EBV, Lymphoma, Lymphoproliferative disorder

## Abstract

**Background:**

Ruxolitinib, a novel inhibitor of Janus kinases 1 and 2, was recently approved for the treatment of myelofibrosis but, recently, attention has been drawn to potential side effects and especially opportunistic infections and virus reactivations. EBV reactivation has not previously been reported to occur in association with Ruxolitinib.

**Case presentation:**

We report a case of a 57 year old female with post-polycythemic myelofibrosis who was treated with Ruxolitinib. Approximately 9 weeks later she presented with a rapidly fatal, suspected EBV driven lymphoproliferative disorder in the CNS.

**Conclusions:**

Our report further underlines that patients treated with Ruxolitinib should be monitored closely for reactivations of opportunistic pathogens and viral infections.

## Background

Ruxolitinib was recently approved for the treatment of myelofibrosis and numerous studies are underway examining its effect in other myeloproliferative neoplasms. This novel inhibitor of Janus kinases 1 and 2 has shown promising effects in reducing constitutional symptoms and splenomegaly in patients with myelofibrosis and more recently was also shown to increase overall survival [1, 2]. Recently, attention has been drawn to potential side effects of Ruxolitinib and especially opportunistic infections and viral reactivations [3–9]. Here we report on a patient with a rapidly fatal, suspected EBV driven lymphoproliferative disorder in the CNS developing shortly after administration of Ruxolitinb.

## Case presentation

A 57 year old Caucasian woman with a history of Polycythemia vera (PV) presented to our institution with progressive debilitating constitutional symptoms. She had received her diagnosis 25 years ago and underwent splenectomy 15 years earlier after a splenic vein thrombosis. Three years earlier she was diagnosed with focal segmental glomerulosclerosis (FSGS) with a progressive renal failure, presently with an estimated glomerular filtration rate according to plasma Iohexol clearance of 43 mL/min/1,73 m^2^ and a nephrotic syndrome. Earlier, her FSGS was treated with Prednisolone, Tacrolimus and Adrenocorticotrophic hormone (ACTH) but currently she was not under active treatment. Throughout the years she received numerous treatment modalities for her PV including phlebotomy, 32P, Hydroxyurea and Anagrelide. At the time of presentation progressive debilitating constitutional symptoms in the form of fatigue, low grade fever and weight loss had developed. A diagnosis of post-polycythemic myelofibrosis was made on a bone marrow biopsy. She was treated with pegylated interferon alfa-2a but shortly after her second injection developed severe headache that required hospital admission. CT and MRI of the brain were normal. Lumbar puncture was not performed. Progressive proteinuria was noted and the patient was treated with Prednisolone 30 mg daily. The pegylated interferon alfa 2a treatment was stopped and her headache gradually disappeared. Three weeks later, treatment with Ruxolitinib was started at a dose of 10 mg bid and the patient had a partial resolution of symptoms. Approximately 9 weeks after the beginning of Ruxolitinib therapy the patient was admitted, now for three grand mal seizures.

**Fig. 1:**
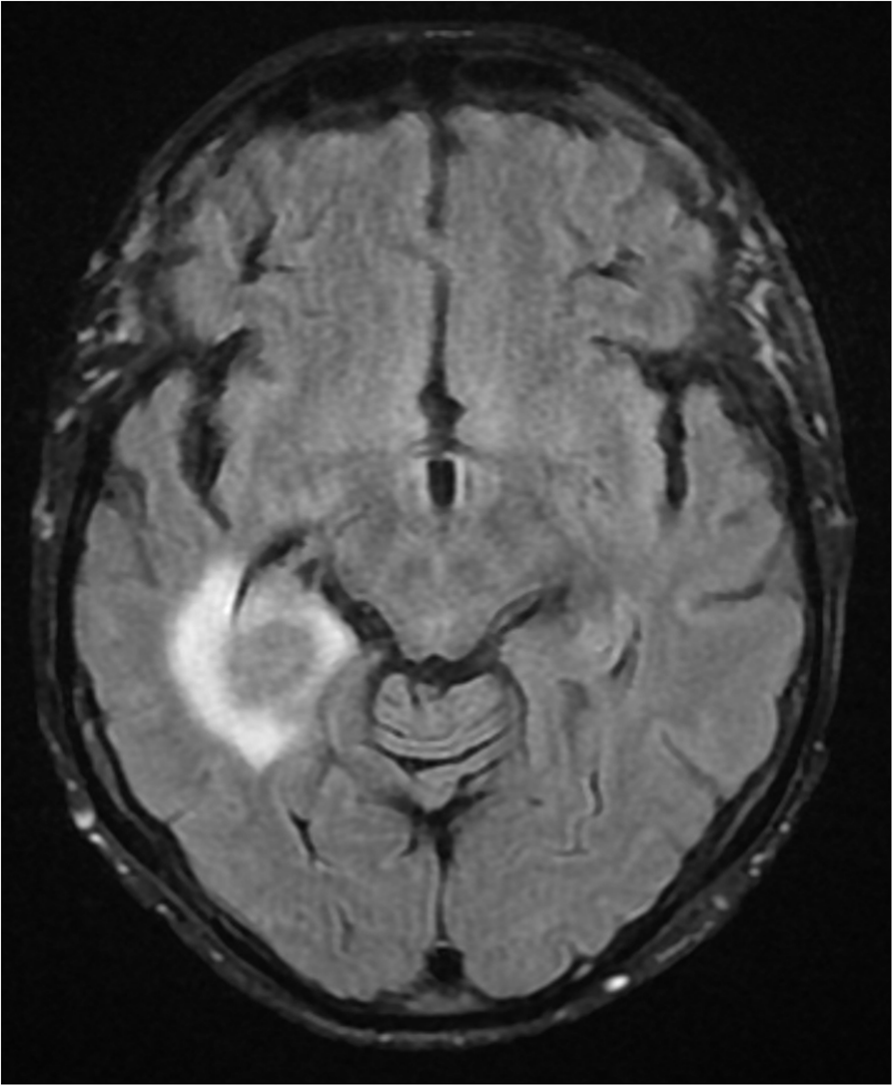
MRI of the head showing ring enhanced lesion in the right temporal lobe with surrounding edema at the time of diagnosis

**Fig. 2:**
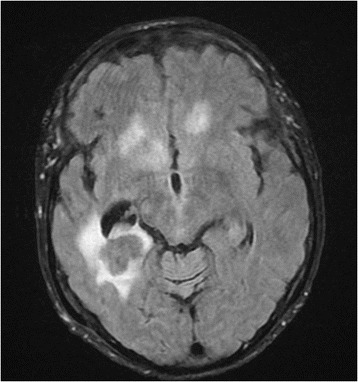
MRI at time of progression 4 weeks later

On diffusion-weighted MRI scan of the brain there was a 2 cm ring lesion in the right temporal lobe with restricted diffusion and surrounding edema (Fig. 1). The radiographic appearance was highly suspicious of a CNS lymphoma. Retrospective examination of MRI taken 11 weeks earlier showed no signs of tumor. PET-CT showed 18 F-FDG lesion uptake but no signs of disseminated disease. Cerebrospinal fluid (CSF) analysis showed marginally elevated mononuclear cells (11 × 10^6^/mL) but flow cytometry analysis and cytology of the CSF were negative. Bacterial and mycobacterial cultures were negative. However, PCR analysis of CSF was positive for Epstein-Barr virus with 100 000 copies/mL while only mildly positive in serum with <250 copies/mL. PCR for JC, CMV, HHV6, HSV and VZV virus in the CSF were negative. A diagnosis of CNS lymphoproliferative disorder was suggested and Ruxolitinib was stopped. No biopsy was made. Because of co-morbidities the patient was deemed not to be a candidate for aggressive chemotherapy and palliative CNS radiotherapy, 24 Gy in 12 daily fractions, was commenced and she was treated concurrently with Rituximab and Temozolamide. Her condition worsened quickly thereafter and a repeated MRI showed a progressive disease (Fig. 2), now with multiple metastases in the brain and a midline shift. The patient passed away 5 weeks after her diagnosis. An autopsy was not performed.

## Discussion

In the earlier studies on the efficacy and safety of Ruxolitinib the major reported side effects were dose-related hematologic toxicity with anemia, neutropenia and thrombocytopenia. Herpes Zoster reactivation was described and awareness for this is recommended as is screening for latent tuberculosis [2]. Recently, published reports have described viral reactivations of Hepatitis B [3, 5] toxoplasma chorioretinitis [4], cryptococcus neoformans [8], mucormycosis, herpes simplex [6] and pneumocystis jiroveci [9] associated with the use of Ruxolitinib.

Studies have been conducted where the immunosuppressive effects of Ruxolitinib have been evaluated. A phase 1/2 study showed a marked decrease in pro-inflammatory cytokines in patients receiving Ruxolitinib [10] which likely plays a role in the favorable effect of the drug in reducing myelofibrosis related symptoms. A pre-clinical model showed reduced dendritic cell function after the administration of Ruxolitinib resulting in an impaired CD4^+^ and CD8^+^ T cell priming in vitro and in vivo [11]. Another study in patients receiving Ruxolitinib showed a marked and long-lasting decrease in regulatory T-cells, which are known to have a protective role against viral pathogens [12].

To our knowledge this is the first case describing EBV driven suspected lymphoproliferative disorder during Ruxolitinib therapy. Although no biopsy was made, the radiographic finding of a cerebral ring enhanced lesion with an avid 18 F-FDG uptake in a patient with a high copy number of EBV in the CSF and the virtual absence of EBV transcripts in serum, makes the suggested diagnosis highly plausible. Other differential diagnosis of cerebral ring enhanced lesions including metastases, abscesses or tuberculosis seem less likely in this case. Ruxolitinib may have, in conjunction with other immunosuppressive factors such as asplenia and long term steroid treatment, led to EBV reactivation in this patient who was seropositive for EBV (IgG but not IgM) before treatment initiation. The fact that the patient did undergo an MRI of the brain, that was normal, only 3 weeks prior to initiation of Ruxolitinib therapy strongly suggests that the disorder developed after the beginning of Ruxolitinib therapy.

## Conclusions

We describe a patient with post-polycythemic myelofibrosis who was treated with Ruxolitinib. Nine weeks later she presented with a rapidly fatal EBV driven suspected CNS lymphoproliferative disorder which was refractory to radiotherapy, Rituximab and Temozolamid. Our report further underlines that patients treated with Ruxolitinib should be monitored for reactivation of opportunistic pathogens and viral infections, as recently suggested [13].

### Consent

Written informed consent was obtained from the relatives of our patient for publication of this Case report. A copy of the written consent is available for review by the Editor-in-Chief of this journal.

## Abbreviations

18 F-FDG: 18 F-Fluorodeoxyglucose
ACTH: Adrenocorticotrophic hormone
CNS: Central nervous system
CSF: Cerebrospinal fluid
CT: Computed tomography
EBV: Epstein Barr virus
FSGS: Focal segmental glomerulosclerosis
MRI: Magnetic resonance imaging
PCR: Polymerase chain reaction
PET: Positron emission tomography
